# Focal adhesion kinase is required for actin polymerization and remodeling of the cytoskeleton during sperm capacitation

**DOI:** 10.1242/bio.017558

**Published:** 2016-07-11

**Authors:** Ana L. Roa-Espitia, Eva R. Hernández-Rendón, Rafael Baltiérrez-Hoyos, Rafaela J. Muñoz-Gotera, Antonieta Cote-Vélez, Irma Jiménez, Humberto González-Márquez, Enrique O. Hernández-González

**Affiliations:** 1 Departamento de Biología Celular, Centro de Investigación y Estudios Avanzados del Instituto Politécnico Nacional, México D.F. 07360, México; 2 Posgrado en Biología Experimental, Universidad Autónoma Metropolitana-Iztapalapa, México D.F. 09349, México; 3 Universidad Autónoma Benito Juárez de Oaxaca, Facultad de Medicina y Cirugía, Oaxaca, Oaxaca 68120, México; 4 Facultad de Ciencias Veterinarias, Universidad de Zulia, Maracaibo 4012, Venezuela; 5 Departamento de Genética del Desarrollo y Fisiología Molecular, Instituto de Biotecnología, Universidad Nacional Autónoma de México 62210, Cuernavaca, México; 6 Departamento de Ciencias de la Salud, Universidad Autónoma Metropolitana-Iztapalapa, México D.F. 09349, México

**Keywords:** Acrosome reaction, Capacitation, Cytoskeleton, Protein tyrosine (Tyr) phosphorylation, Calcium PyK2

## Abstract

Several focal adhesion proteins are known to cooperate with integrins to link the extracellular matrix to the actin cytoskeleton; as a result, many intracellular signaling pathways are activated and several focal adhesion complexes are formed. However, how these proteins function in mammalian spermatozoa remains unknown. We confirm the presence of focal adhesion proteins in guinea pig spermatozoa, and we explore their role during capacitation and the acrosome reaction, and their relationship with the actin cytoskeleton. Our results suggest the presence of a focal adhesion complex formed by β1-integrin, focal adhesion kinase (FAK), paxillin, vinculin, talin, and α-actinin in the acrosomal region. Inhibition of FAK during capacitation affected the protein tyrosine phosphorylation associated with capacitation that occurs within the first few minutes of capacitation, which caused the acrosome reaction to become increasingly Ca^2+^ dependent and inhibited the polymerization of actin. The integration of vinculin and talin into the complex, and the activation of FAK and paxillin during capacitation, suggests that the complex assembles at this time. We identify that vinculin and α-actinin increase their interaction with F-actin while it remodels during capacitation, and that during capacitation focal adhesion complexes are structured. FAK contributes to acrosome integrity, likely by regulating the polymerization and the remodeling of the actin cytoskeleton.

## Abstract (precis)

**Summary:** We describe the role of FAK and focal adhesion proteins in capacitation, acrosome reaction, polymerization and remodeling of actin cytoskeleton, and how inhibition of FAK affects sperm physiology.

## INTRODUCTION

Successful fertilization of the oocyte requires that spermatozoa undergo physiological and biochemical changes that are known as capacitation: increased metabolism, membrane fluidity, intracellular Ca^2+^ concentration, membrane hyperpolarization, intracellular cAMP concentration, protein tyrosine phosphorylation (Tyr phosphorylation), concentration of reactive oxygen species, actin polymerization, and motility (Aitken and Nixon, 2013; Bailey, 2010). *In vivo* spermatozoa are capacitated by interacting with environmental stimuli in the female reproductive tract prior to encountering oocytes. One of these stimuli requires that spermatozoa interact with several extracellular matrices (ECMs) that are composed of a variety of glycoproteins, such as laminin, fibronectin, and collagen type IV, found in epithelial cells of the caudal isthmus or cumulus oophorus (Makrigiannakis et al., 2009; Sutovsky et al., 1995; Thys et al., 2009). Carbohydrates, glycoproteins, epithelial cadherin, and integrins are components of sperm cells that are known to modulate adhesion and binding during reproductive processes, such as spermatozoa-oviduct adhesion and spermatozoa-oocyte interactions (Barraud-Lange et al., 2007; Boissonnas et al., 2010; Caballero et al., 2014; Talevi and Gualtieri, 2010; Thys et al., 2009).

The remodeling of the actin cytoskeleton in mammalian spermatozoa is a process that involves actin polymerization and is necessary for the acrosome reaction (AR) to function normally, and for sperm to achieve adequate motility (Azamar et al., 2007; Brener et al., 2003; Itach et al., 2012). Studies have demonstrated that an increase in F-actin during capacitation depends upon the activation of gelsolin. This actin-severing protein associates with phosphatidylinositol-4, bisphosphate (PIP2) (Finkelstein et al., 2010) which is important to motility because reduced synthesis of PIP2 inhibits actin polymerization, consequently inhibiting sperm motility (Finkelstein et al., 2013). Furthermore, inhibition of actin polymerization is known to diminish the ability of spermatozoa to fertilize the oocyte (Brener et al., 2003; Rogers et al., 1989; Sanchez-Gutierrez et al., 2002), however a detailed understanding of how actin polymerization is regulated during capacitation remains unknown.

Mouse and bovine spermatozoa have been shown to express the integrins α6β1, α5β1, and αvβ3, and the proteins involved in the adhesion and fusion of spermatozoa with oocytes (Barraud-Lange et al., 2007; Boissonnas et al., 2010; Thys et al., 2009). These findings suggest that focal adhesion proteins are present in mammalian spermatozoa, and that they may be involved in their physiological processes, including capacitation, the AR, and motility.

Integrins are heterodimeric transmembrane proteins involved in cellular processes, such as cell-cell adhesion or cell-ECM interactions. It is well established that integrins mediate interactions between the actin cytoskeleton and ECM proteins, which imply dynamic remodeling of this cytoskeleton, influencing cellular survival: adhesion of cells to the ECM promotes cell survival, while their detachment can induce apoptosis (Paoli et al., 2013). These processes occur through a variety of signaling mechanisms where the formation of focal adhesions has a pivotal role (Reddig and Juliano, 2005). Structural modifications of focal adhesions require the assistance of accessory proteins, such as focal adhesion kinase (FAK), paxillin, vinculin, α-actinin, filamin, talin, and tensin to mediate the interaction between the EMC and the actin cytoskeleton. FAK, proline-rich tyrosine kinase-2 (PyK2) and integrin-linked kinases are important protein tyrosine kinases associated with focal adhesion complexes, and they are activated by calcium or when integrins engage with ECM proteins (Hall et al., 2011).

Activation of FAK initiates a number of biological processes, including cell attachment, migration, invasion, proliferation, and survival. The cytoplasmic tail of β-integrin (β1, β2, and β3) facilitates FAK activation by means of an undefined mechanism that involves integrin clustering, FAK autophosphorylation at Tyr397, and the mechanical linkage of integrins to the actin cytoskeleton. In its activated state FAK functions as an adaptor protein to recruit other focal contact proteins or their regulators, which affects the assembly or disassembly of cell-cell (cadherin-based) or cell-ECM focal contacts (Schaller, 2010). FAK also functions as a scaffold to organize signaling proteins within focal adhesion complexes. FAK can influence the activity of the proteins that regulate actin cytoskeleton assembly, such as Rho-family GTPases (RhoA, Rac, and Cdc42). Specifically, FAK facilitates the localization and cyclic activation of guanine nucleotide exchanger factors and GTPase-activating proteins that regulate the activity of the Rho protein. Thus, FAK has an important role in processes regulated by the Rho proteins (Tomar and Schlaepfer, 2009).

FAK (also known as protein tyrosine kinase 2, PTK2) and the closely related PyK2 (also known as protein tyrosine kinase 2b, PTK2b) are non-receptor tyrosine kinases with an important role in the phosphorylation of downstream substrates for the transmission of cellular signals. Both proteins act as scaffolds and are integral to the assembly of different signaling complexes (Hall et al., 2011); however, only PyK2 has been found in mammalian spermatozoa (Battistone et al., 2014; Chieffi et al., 2003; Gonzalez-Fernandez et al., 2013; Rotfeld et al., 2014). In mouse spermatozoa, PyK2 is located in the acrosome region and principally regulates the Tyr phosphorylation that occurs during capacitation. Together with calcium/calmodulin-dependent protein kinase II, PyK2 regulates Tyr phosphorylation in stallion spermatozoa (Gonzalez-Fernandez et al., 2013). PyK2 has also been shown to contribute to the activation of phosphatidylinositol-3-kinase, a kinase implicated in the induction of the AR in bovine spermatozoa (Rotfeld et al., 2014). Meanwhile, in human spermatozoa PyK2 is considered to be an intermediary component of Ca^2+^ signaling between PKA-mediated and Tyr phosphorylation that is required for achieving functional human sperm capacitation (Battistone et al., 2014).

In this work, we postulate that the presence of integrins in mammalian spermatozoa suggests the presence of focal adhesion proteins that likely participate in various sperm processes through the activation of FAK during capacitation. We aim to evidence the presence of focal adhesion proteins, localize them in guinea pig spermatozoa, and determine whether they form complexes. Next, we seek to understand whether FAK is activated throughout capacitation and to investigate the consequences of inhibiting FAK on capacitation and the AR. Finally, we study the relationship between focal adhesion proteins and the actin cytoskeleton, and we examine the effects of inhibiting FAK on actin polymerization during capacitation.

## RESULTS

### Focal adhesion proteins are expressed in guinea pig spermatozoa

**Fig. 1.**
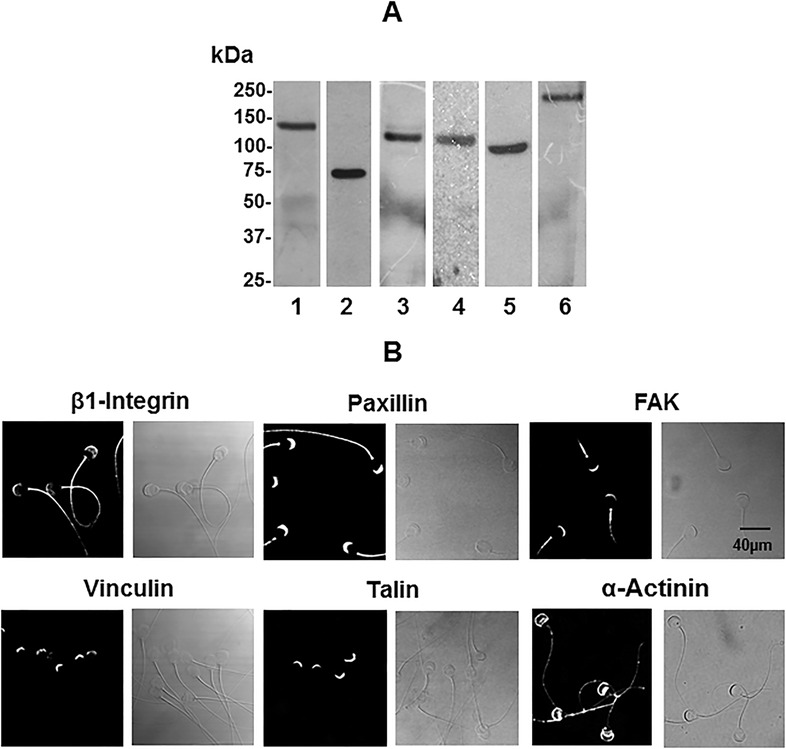
**Focal adhesion proteins are expressed in guinea pig spermatozoa.** (A) For the detection of focal adhesion proteins in guinea pig spermatozoa, 100 µg of whole sperm extracts were resolved by SDS-PAGE, transferred to nitrocellulose membranes, and the proteins were detected using specific antibodies. (1) β-integrin, (2) paxillin, (3) FAK, (4) vinculin, (5) α-actinin, and (6) talin. (B) Immunocytochemistry assays were performed to determine the localization of focal adhesion proteins in guinea pig spermatozoa. Left panels correspond to the localization of β-integrin, paxillin, FAK, vinculin, α-actinin, and talin. Right panels correspond to the bright field images. Images are representative of three independent experiments.

The presence of β1-integrin in human, bovine, and mouse spermatozoa (Barraud-Lange et al., 2007; Boissonnas et al., 2010; Thys et al., 2009) and the presence of phosphorylated FAK in horse spermatozoa (Gonzalez-Fernandez et al., 2013) motivated us to find evidence of the existence of FAK and other focal adhesion proteins in a mammalian model. We assayed the presence of focal adhesion proteins in guinea pig spermatozoa and evaluated the expression of these proteins by immunoblot analysis in whole guinea pig sperm lysates. Specific bands corresponding with relative molecular weight (Mr) of ∼130, 70, 125, 120, 100, and 230 kDa were detected for β1-integrin, paxillin, FAK, vinculin, α-actinin, and talin, respectively (Fig. 1A). Because most of these proteins have not previously been detected in mammalian spermatozoa, we validated the antibodies used in this study by carrying out a positive control with the breast cancer cell line MDA-MB-231 and with human and mouse spermatozoa. All six proteins had similar Mr for the corresponding antigen and to those described for sperm proteins (Fig. S2A, Fig. S1A,B, respectively). These results confirm that the proteins we found in guinea pig spermatozoa are the focal adhesion proteins β1-integrin, paxillin, FAK, vinculin, α-actinin, and talin, and that they are also expressed in the spermatozoa of other species.

We used immunocytochemistry analysis to determine that all six focal adhesion proteins localize in the acrosome of guinea pig spermatozoa (Fig. 1B). More specifically, β1-integrin and paxillin were localized along the flagellum, while FAK was detected only in the mid-piece (Fig. 1B). Note that their localization patterns were similar in non-capacitated and capacitated spermatozoa. In contrast, α-actinin was localized mainly to the periphery of the acrosomal region, with only a faint presence along the flagella (Fig. 1B). Similar results were obtained for mouse and human spermatozoa (Fig. S1C,D).

### Focal adhesion proteins form a protein complex that is structured during capacitation

**Fig. 2.**
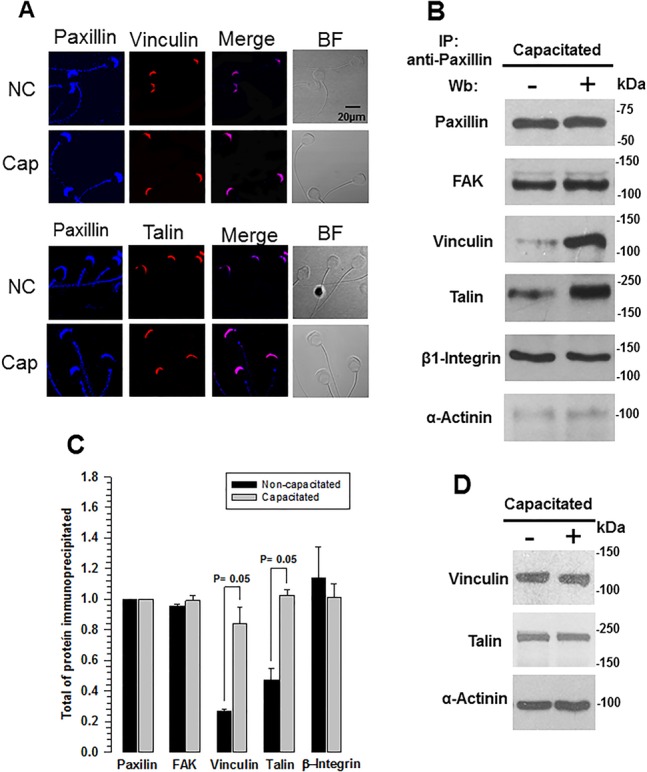
**Talin and vinculin are associated with the focal adhesion complex during capacitation.** (A) To determine the changes undergone by vinculin and talin in the focal adhesion complex, co-localization of vinculin and talin (red) with paxillin (blue) was visualized by immunocytochemical assays in formaldehyde-fixed non-capacitated (NC) and capacitated (Cap) guinea pig spermatozoa. The merged (pink) and bright field (BF) images are also shown. Images are representative of three independent experiments. (B) Co-immunoprecipitation assays of paxillin with the focal adhesion proteins were performed to determine changes in the complex. Total sperm extracts were immunoprecipitated from non-capacitated and capacitated guinea pig spermatozoa using the anti-paxillin antibody. The precipitated proteins were subject to SDS-PAGE and western blot analysis was performed using anti-paxillin, anti-FAK, anti-β-integrin, anti-vinculin, anti-talin, and anti-α-actinin antibodies. The image is representative of five independent experiments. (C) Densitometric analysis of co-immunoprecipitated focal adhesion proteins. The results are expressed as the ratio *N*/No, where *N* is the total amount of each co-immunoprecipitated protein and No is the total amount of paxillin immunoprecipitated (mean±s.e.m., *n*=3 independent experiments). (D) Western blot showing that the amount of vinculin, talin, and α-actinin did not change during capacitation. Images are representative of three independent experiments.

Considering that all focal adhesion proteins localized in the acrosome region, we hypothesized that these proteins form complexes and might undergo changes during capacitation. To test this hypothesis, we performed co-localization and co-immunoprecipitation assays in non-capacitated and capacitated guinea pig spermatozoa. Paxillin is a scaffolding protein containing multiple domains that mediate protein-protein interactions, including domains for β1-integrin, FAK, vinculin, and talin (Brown and Turner, 2004), for this reason we used paxillin as reference protein. Assays of co-localization performed in non-capacitated spermatozoa showed that paxillin co-localized with FAK and β1-integrin in the acrosome region and along the mid-piece, these patterns remained for capacitated spermatozoa (Fig. S3A). We observed far less co-localization of paxillin with vinculin and talin in non-capacitated spermatozoa, which increased during capacitation, particularly in the apical region of the acrosome (Fig. 2A). Stronger co-localizations throughout the acrosome in capacitated spermatozoa suggest focal adhesion complexes in spermatozoa undergo changes during capacitation. To test this hypothesis we conducted co-immunoprecipitation assays to find that paxillin immunoprecipitated from whole-cell extracts of non-capacitated and capacitated spermatozoa, immunoblots for each focal adhesion protein were also performed. Densitometric analysis of the proteins showed that paxillin co-immunoprecipitated with similar amounts of FAK and β1-integrin for both non-capacitated and capacitated spermatozoa (Fig. 2B,C); however, in non-capacitated spermatozoa significantly less vinculin and talin co-immunoprecipitated, and in capacitated spermatozoa comparable amounts of FAK, β1-integrin, vinculin, and talin co-immunoprecipitated with paxillin (Fig. 2B,C). In both non-capacitated and capacitated spermatozoa, only a small amount of α-actinin co-immunoprecipitated with paxillin (Fig. 2B,C). We performed an additional immunoblot to eliminate differences in the amounts of vinculin, talin, and α-actinin that co-immunoprecipitated as the cause of changes in these proteins during capacitation. We found similar amounts of these three proteins in both non-capacitated and capacitated spermatozoa (Fig. 2D). These results suggest that the focal adhesion proteins found in spermatozoa form complexes during capacitation.

### FAK and paxillin phosphorylation increases during capacitation

**Fig. 3.**
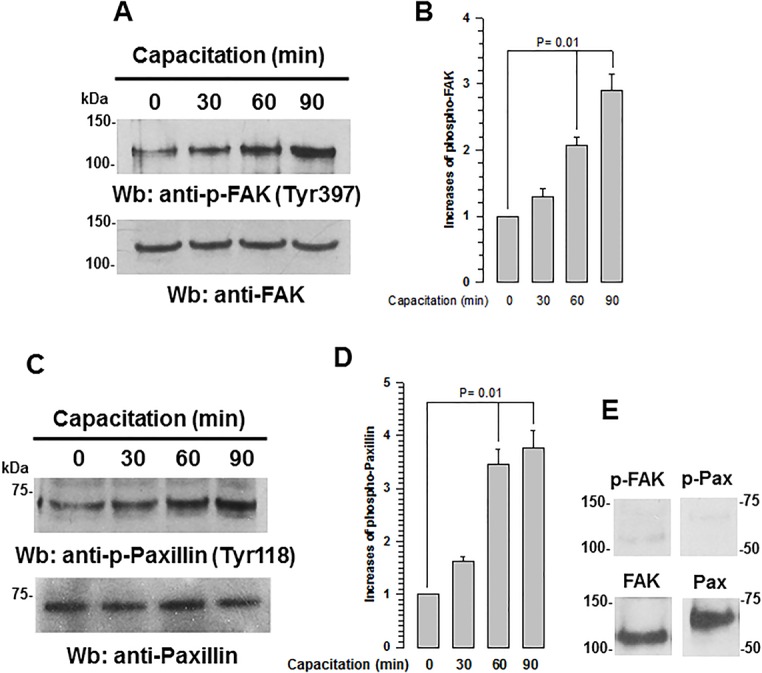
**FAK and paxillin phosphorylation increase during capacitation.** To determine the phosphorylation state of FAK and paxillin, total extracts from capacitated guinea pig spermatozoa (0-90 min) were subjected to SDS-PAGE and analyzed by western blotting. (A) Upper panel: p-FAK detected with anti-p-FAK (Tyr397). Lower panel: total FAK detected with anti-FAK antibody. (B) Increase of p-FAK during capacitation (mean±s.e.m., *n*=3 independent experiments). (C) Upper panel: p-paxillin detected with anti-p-paxillin (Tyr118) antibody; lower panel: total paxillin detected with anti-paxillin antibody. (D) Increase of p-paxillin during capacitation (mean±s.e.m., *n*=3 independent experiments). (E) Upper panel: western blot showing the effect of PF573228 on the phosphorylation of FAK (Tyr397) and paxillin (Tyr118) detected with the respective anti-phosphorylated protein antibody; lower panel: total FAK and paxillin detected with anti-FAK and anti-paxillin antibodies. Images are representative of three independent experiments.

We studied the phosphorylation status of FAK and paxillin during capacitation in guinea pig spermatozoa. In non-capacitated spermatozoa, basal phosphorylation was detected for FAK at Tyr397 (p-FAK) and paxillin at Tyr118 (p-Pax) (Fig. 3). A significant increase in phosphorylation occurred 60 min after capacitation (Fig. 3A,C), both reaching a maximum 90 min after capacitation (Fig. 3B,D). To determine whether the increases in phosphorylation were specific, spermatozoa were capacitated in the presence of PF537228 (5 µM), a competitive inhibitor of ATP with high specificity for FAK, preventing FAK phosphorylation at Tyr397 and subsequently its activation (Slack-Davis et al., 2007). The immunoblot analysis of p-FAK and p-Pax showed that PF537228 abolished the phosphorylation of both proteins (Fig. 3E).

**Fig. 4.**
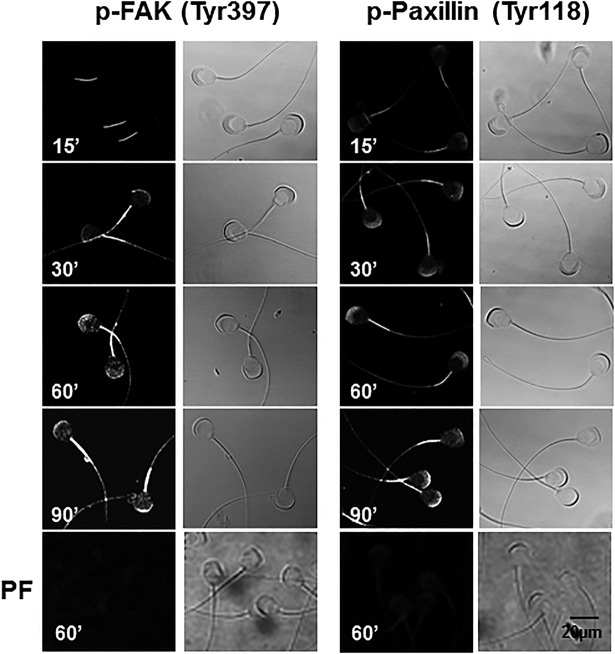
**FAK and paxillin phosphorylation increase in the acrosome region and mid-piece during capacitation.** To determine where in the sub-cellular sperm region phosphorylation increases during capacitation, capacitated (15-90 min) guinea pig spermatozoa were fixed with formaldehyde and phosphorylation was analyzed using anti-p-FAK (Tyr397) or anti-p-paxillin (Tyr118) antibodies. To determine whether staining was specific, spermatozoa were treated with PF573224 (PF). The images on the right correspond to the phase contrast images. Images are representative of three independent experiments.

Because we identified FAK and paxillin in both the acrosome region and the mid-piece, we investigated the subcellular presence of these phosphorylated proteins. In non-capacitated spermatozoa, p-FAK and p-Pax were primarily localized in the mid-piece and a pale fluorescence indicated a weak presence in the acrosomal region (Fig. 4). After 30 min of incubation under capacitation conditions, fluorescence increased in the mid-piece and acrosomal region, reaching a maximum after 90 min of capacitation (Fig. 4). These results indicate that FAK and paxillin have a basal level of activity in non-capacitated spermatozoa that increases during capacitation. To determine whether increased staining of p-FAK and p-Pax during capacitation was specific, spermatozoa were capacitated in the presence of PF573228. After 60 min of capacitation, spermatozoa displayed no fluorescence for p-FAK or p-Pax (Fig. 4PF).

### FAK inhibition does not alter the phosphorylation of proteins at tyrosine residues during capacitation

**Fig. 5.**
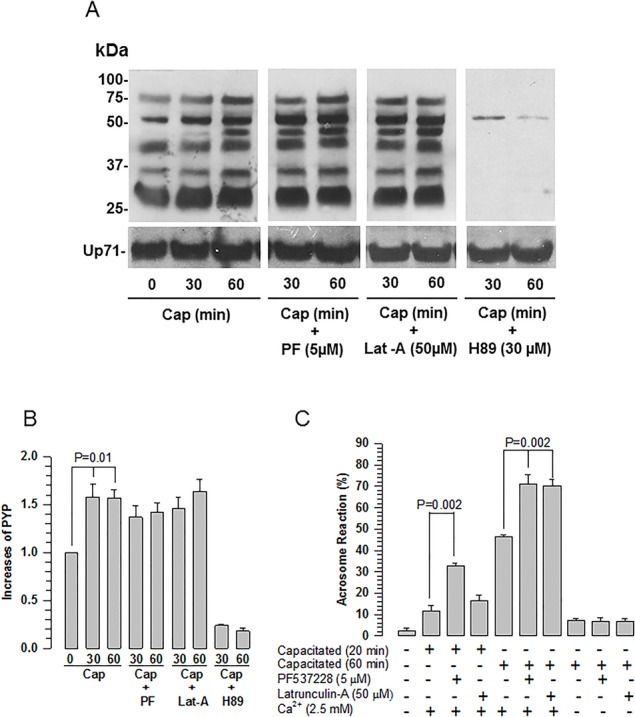
**Effects of PF573228 and Latrunculin-A on capacitation and the AR.** Guinea pig spermatozoa were capacitated at different times in the presence of the inhibitors of FAK [PF573228 (PF)], actin polymerization [Latrunculin A (Lat-A)], or PKA (H89) at the indicated concentrations. (A) For the detection of Tyr phosphorylation, 100 µg of whole sperm extracts were resolved by SDS-PAGE, transferred to nitrocellulose membranes, and the proteins were detected using a specific antibody against p-Tyr. The image represents five independent experiments of each treatment. Cap: capacitated. (B) Densitometric analysis of the Tyr phosphorylation increases during capacitation (Cap) (mean±s.e.m., *n*=3 independent experiments). (C) To determine the effect of PF573228 or Lat-A on the AR, spermatozoa were capacitated in the presence of these drugs. The AR was evaluated in spermatozoa capacitated in the presence or absence of Ca^2+^ (mean±s.e.m., *n*=3 independent experiments).

One important feature of capacitation is the increase in Tyr phosphorylation (Visconti et al., 2011). Because it was recently proposed that focal adhesion kinase I (PyK2) is involved the regulation of Tyr phosphorylation (Battistone et al., 2014), we evaluated the effect of inhibiting FAK on Tyr phosphorylation. Using an antibody against phosphorylated Tyr (p-Tyr), we found only a basal level of Tyr phosphorylation in non-capacitated spermatozoa that increased as a function of capacitation, an increase that was inhibited by H-89, an antagonist of PKA (Fig. 5A,B). When spermatozoa were capacitated in the presence of the FAK phosphorylation inhibitor PF573228, Tyr phosphorylation proceeded similarly to when spermatozoa were capacitated in the absence of PF573228 (Fig. 5A,B). We used the chlortetracycline test (Ward and Storey, 1984) to assess the percentage of spermatozoa capacitated in the assays described above. The probe showed a high percentage of pattern B (spermatozoa capacitated) at 30 and 60 min (53.07±5.82 and 70.70±8.05, s.d., *n*=3, respectively). In the presence of PF537228 (5 μM) during capacitation, spermatozoa exhibited a significantly low percentage of pattern B (12.86±6.89 and 15.24±9.73, s.d., *n*=3, respectively) and a high percentage of pattern AR (acrosome-reacted spermatozoa) (63.27±4.54 for 30 min and 70.55±4.84 for 60 min, s.d., *n*=3).

**Fig. 6.**
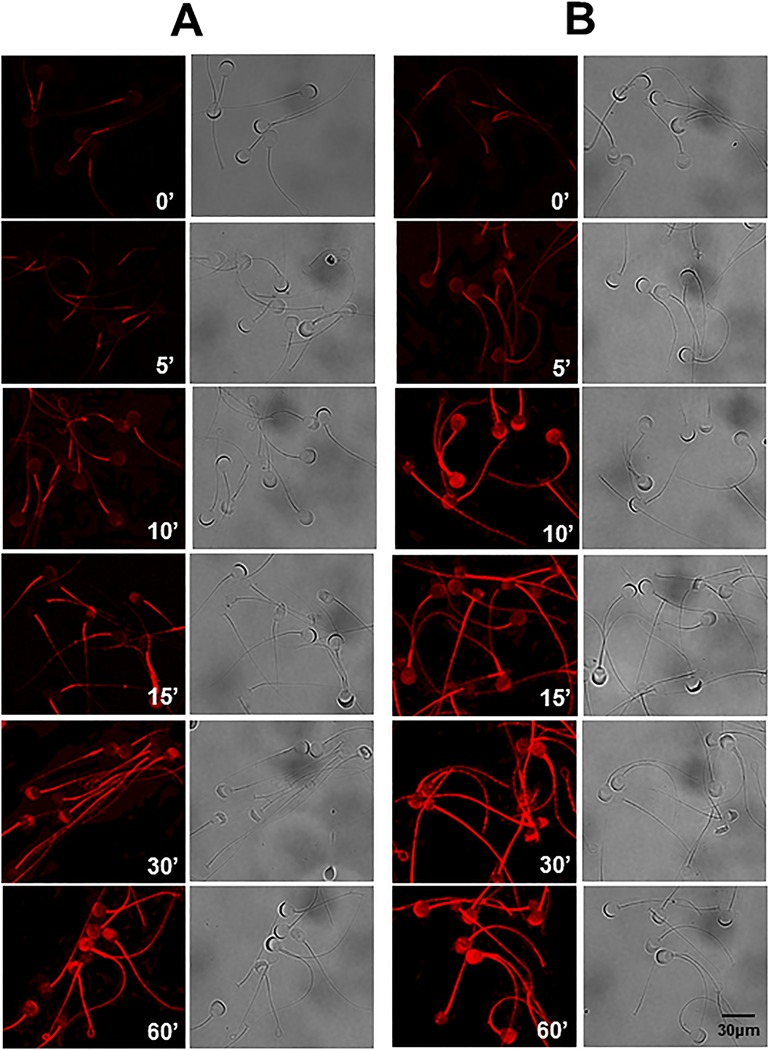
**Effect of PF537228 on protein tyrosine phosphorylation.** Spermatozoa were incubated under capacitation conditions in presence or absence of the FAK inhibitor PF537228 for different times and then fixed and used for the immunolabeling assay. To detect the proteins Tyr phosphorylation, we used a specific antibody anti-p-Tyr. (A) Immunostaining of Tyr phosphorylation in capacitated spermatozoa. (B) Immunostaining of Tyr phosphorylation in spermatozoa incubated under capacitation conditions in the presence of PF537228 (5 μM). Images on the left correspond to phosphotyrosine labeling, and images on the right correspond to the bright field of those on the left.

To confirm that spermatozoa incubated in presence of PF537228 were subject to Tyr phosphorylation, we analyzed the Tyr phosphorylation state during capacitation by immunofluorescence. Non-capacitated spermatozoa showed a pale fluorescence in their mid-piece, which gradually increased during the course of capacitation. After 30 min of capacitation, fluorescence was evident along the flagellum and in the acrosome, while the highest fluorescence was observed at 60 min (Fig. 6A). Spermatozoa incubated under capacitation conditions and in the presence of PF537228 displayed a high fluorescence pattern along their flagella and in their acrosomes during the first 10 min of incubation. Fluorescence intensity was higher than that exhibited by spermatozoa capacitated for 10 min and similar to that exhibited by spermatozoa capacitated for 30 min in the absence of PF537228. Spermatozoa incubated under capacitation conditions and in the presence of PF537228 showed the highest fluorescence at 30 min of incubation (Fig. 6B). These results suggest that spermatozoa incubated under capacitation conditions and in the presence of PF537228 experienced an early capacitation and consequently an anticipated AR.

### FAK inhibition induces calcium-dependent AR

To determine the effect of FAK inhibition on the AR, spermatozoa were capacitated in the presence of PF573228 (5 µM) and the AR was quantified. As early as 20 min into incubation under capacitation conditions, the AR significantly increased for spermatozoa treated with PF573228 compared to untreated spermatozoa. After 60 min, the AR increased for both treated and untreated groups, although a similar difference between the samples remained evident (Fig. 5C). To define the effect of PF573228 on AR, we performed similar experiments using Latrunculin A (Lat-A), an actin polymerization inhibitor. After 20 min of incubation under capacitation conditions, the AR proceeded similarly for spermatozoa treated or untreated with Lat-A; however, after 60 min the AR increased significantly in spermatozoa treated with Lat-A, approaching values similar to those treated with PF573228 (Fig. 5C).

The AR depends on the extracellular concentration of Ca^2+^; therefore, the AR is blocked when spermatozoa are capacitated in the absence of Ca^2+^ (Darszon et al., 2011). To distinguish whether the decreased acrosome function induced by PF573228 or Lat-A is a true AR, spermatozoa were capacitated in the absence of Ca^2+^. After 60 min of capacitation, spermatozoa capacitated in the presence PF573228 or Lat-A displayed a significantly reduced AR (Fig. 5C). These results suggest that the inhibition of FAK by PF573228 induces the AR, probably as a consequence of changes to the actin cytoskeleton and other pathways related to capacitation.

### Inhibiting FAK prevents actin polymerization during capacitation

**Fig. 7.**
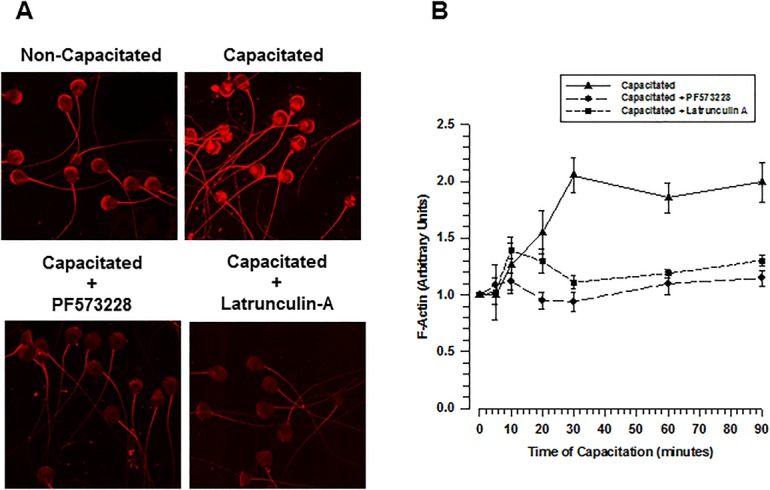
**Both FAK inhibition and Latrunculin-A prevented actin polymerization during capacitation.** (A) To determine the effects of FAK inhibition and Lat-A on actin polymerization, guinea pig spermatozoa were capacitated in the presence or absence of PF573228 (5 µM) or Lat-A (50 µM). F-actin was stained using TRITC-labeled phalloidin. Images are representative of three independent experiments. (B) Fluorescence levels of TRITC-labeled phalloidin were quantified using the software Nis 3.1 to define the time course of F-actin during capacitation in the presence or absence of PF573224 or Lat-A (mean±s.e.m., *n*=5 independent experiments).

To determine whether FAK is involved in actin polymerization during capacitation, F-actin was quantified in spermatozoa with an intact acrosome by evaluating the degree of fluorescence emitted by TRITC-phalloidin. During the first 30 min of incubation under capacitation conditions, fluorescence increased, stabilizing after 60 min (Fig. 7A,B). In the presence of PF537228, spermatozoa exhibited no increase in fluorescence after 60 min of capacitation and levels remained similar to those in non-capacitated spermatozoa (Fig. 7B). F-actin was also quantified in spermatozoa capacitated in the presence of Lat-A. A few minutes after the start of capacitation in the presence of Lat-A, an increase in phalloidin staining was followed by a decrease after 10 min of incubation under capacitation conditions, and after 30 min of incubation it returned to levels similar to those of non-capacitated spermatozoa (Fig. 7B). These results suggest the possibility that during capacitation FAK may be important for actin polymerization and cytoskeleton reorganization.

### Focal adhesion complexes interact with F-actin during capacitation

**Fig. 8.**
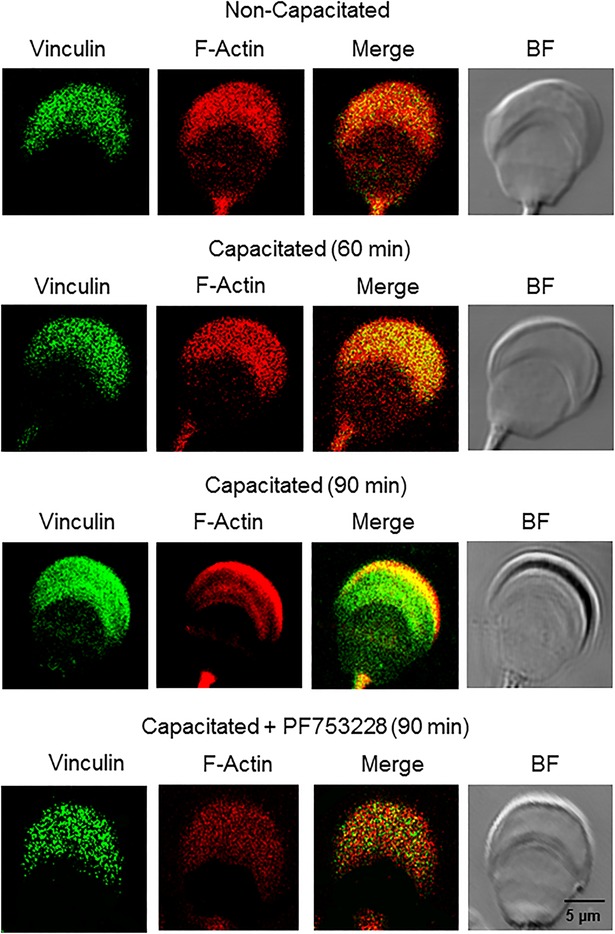
**Vinculin and F-actin co-localize during capacitation.** To determine the interaction of the focal adhesion complex with the actin cytoskeleton, vinculin and F-actin were co-localized in non-capacitated and capacitated guinea pig spermatozoa and in those capacitated in the presence of PF573228. Spermatozoa were fixed with formaldehyde and the proteins were detected using anti-vinculin antibody (green) and TRITC-labeled phalloidin (red). The merged and bright field (BF) images are also shown. Images are representative of three independent experiments.

**Fig. 9.**
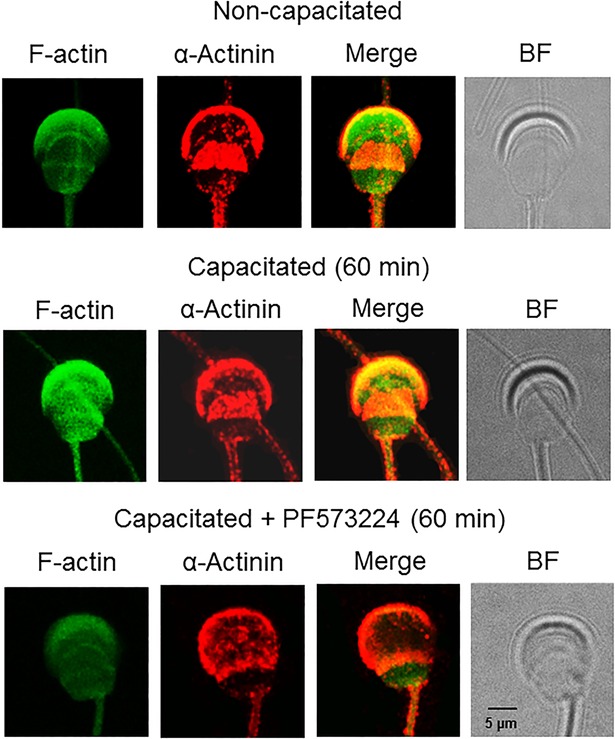
**α-Actinin and F-actin co-localize during capacitation.** To determine the interaction of α-actinin with the actin cytoskeleton, α-actinin and F-actin were co-localized in non-capacitated and capacitated guinea pig spermatozoa and in those capacitated in the presence of PF573228. Spermatozoa were fixed with formaldehyde and proteins were detected using anti-α-actinin antibody (red) and FITC-labeled phalloidin (green). The merged and bright field (BF) images are also shown. Images are representative of three independent experiments.

Focal adhesion complexes are closely linked to F-actin via interactions mediated by proteins such as vinculin or α-actinin (Humphries et al., 2007; Janssen et al., 2006; Kanchanawong et al., 2010). Because we identified both proteins in the acrosomal region, we studied their correlation with F-actin and possible related changes during capacitation. Using confocal microscopy, vinculin and F-actin were identified to co-localize in the acrosomal region of non-capacitated spermatozoa at a low level (Fig. 8). When spermatozoa were capacitated for 60 min vinculin and F-actin co-localized throughout the acrosome, but after 90 min the co-localization was limited to the apical region of the acrosome, the region of the sperm head where most of the F-actin had localized after 60 min (Fig. 8). Similar assays for α-actinin and F-actin showed that the two co-localized along the periphery of the acrosome at a low level in non-capacitated spermatozoa and at a high level in capacitated spermatozoa (Fig. 9). These results indicate that focal adhesion proteins participate in the remodeling of the actin cytoskeleton; therefore, to determine whether FAK is a necessary component of these changes we performed co-localization assays of F-actin/vinculin and F-actin/α-actinin in spermatozoa capacitated in presence of PF537228. Inhibiting FAK had two effects: after 90 min of capacitation, F-actin exhibited a pale fluorescence in spermatozoa capacitated in the presence of PF537228 and low or no co-localization of F-actin with vinculin or α-actinin was observed (Figs 7 and 8). These results suggest that, during capacitation, FAK is involved in the organization and maintenance of the actin cytoskeleton and in actin polymerization.

## DISCUSSION

The integrins α5, α6, αV, β1, and β3 have long been known to exist in mouse and human spermatozoa where they contribute to processes, such as gamete adhesion and fusion (Barraud-Lange et al., 2007; Boissonnas et al., 2010; Thys et al., 2009). The presence of these integrins suggests the existence of focal adhesion proteins in mammalian spermatozoa that could be involved in sperm-specific processes, such as capacitation or the AR. Here, we provide evidence (1) for the formation of focal adhesion complexes during capacitation in guinea pig spermatozoa, (2) that FAK could be related with the normal course of Tyr phosphorylation associated to capacitation, (3) for a relationship between FAK and the polymerization and remodeling of the actin cytoskeleton during capacitation, and (4) that maintaining acrosome integrity during capacitation requires active FAK and the association of focal adhesion complexes with the actin cytoskeleton.

FAK, paxillin, vinculin, talin, and α-actinin are the principal proteins that constitute the focal adhesion complex and are linked to the plasma membrane through the β1- or β3-integrins. Paxillin acts as an important scaffolding protein for focal adhesion complexes, facilitating these interactions (Brown and Turner, 2004). Our results clearly indicate that these proteins are present in guinea pig, mouse and human spermatozoa, where they possibly form two complexes: one composed of β1-integrin, FAK, paxillin, vinculin, α-actinin, and talin localized in the acrosome region and the other composed of β1-integrin, FAK, paxillin and α-actinin localized in the mid-piece (Fig. 1A; Fig. S1C). The presence and similar localization of focal adhesion proteins in the spermatozoa of three species suggests they are important proteins for sperm processes, such as capacitation and the AR. We suggest that the complex found in the acrosome is involved in maintaining its integrity, perhaps via a structured actin cytoskeleton that drives it to remodel during capacitation. Additionally, FAK and the proper functioning of the cytoskeleton may be crucial for maintaining the appropriate time course of the AR. Thus we predict that the complex associated with the mid-piece contributes to the structure of this region where it may also be involved with activation of signal pathways that are related with motility; however, further investigation is necessary to confirm this role.

Our results detailing changes in the co-localization and co-immunoprecipitation of paxillin with vinculin and talin (Fig. 2B) suggest that in non-capacitated spermatozoa, talin and vinculin weakly associate with paxillin and therefore with the focal adhesion complex. In contrast, in capacitated spermatozoa both talin and vinculin were strongly associated with the complex, likely because the focal adhesion complex is stabilized during capacitation. It has been proposed that in nascent adhesion complexes, talin and vinculin are not associated with the complex and are recruited to the complex 60 min after interaction of fibronectin with β1-integrin-containing adhesion complexes, thus forming a mature adhesion complex (Carisey and Ballestrem, 2011; Lawson et al., 2012). In fact, the association of talin with the adhesion complex is a prerequisite for vinculin to interact with the focal adhesion complex (Carisey and Ballestrem, 2011). Recently, the interaction of vinculin with the talin-integrin complex has been shown to drive the recruitment, release, and reorganization of core focal adhesion components (Carisey et al., 2013). These observations support our results and could imply that focal adhesion complexes are nascent in non-capacitated spermatozoa and mature in capacitated spermatozoa. Note that talin and vinculin are associated with the complex at approximately 60 min of capacitation (Fig. 2B), which is similar to that reported for the formation of a mature adhesion complex (Lawson et al., 2012).

The best-characterized FAK phosphorylation event is the autophosphorylation that takes place at Tyr397. It is also known that active FAK phosphorylates paxillin at Tyr31 and Tyr118 and that as a result of this phosphorylation paxillin generates binding sites for the recruitment of numerous signaling molecules related to focal adhesion dynamics (Mitra et al., 2005). Our results show that an increase in the autophosphorylation of FAK is related to an increase in paxillin phosphorylation, which occurs after 60-90 min of capacitation (Figs 3 and 4). Note that the increase in phosphorylation observed in the acrosomal region and the mid-piece correlate with the sites where FAK and paxillin are localized (Figs 1 and 4). This finding suggests that the phosphorylation of FAK and paxillin may facilitate the recruitment of talin and vinculin. In addition, the direct association of vinculin with F-actin indicates that the recruitment of talin and vinculin is related to a major interaction of these focal adhesion complexes with F-actin (Humphries et al., 2007; Janssen et al., 2006). During this interaction, vinculin increasingly co-localizes with F-actin at around 60 min of capacitation. Shortly thereafter, their co-localization becomes limited to the apical acrosome region, suggesting a possible remodeling of the cytoskeleton in the acrosomal region such as forming a barrier between the plasma and the outer acrosome membranes to avoid an early AR. Similar remodeling of the actin cytoskeleton, where a significant amount of F-actin is polarized to the apical acrosome region, has been reported for boar, bovine, guinea pig, and ram capacitated spermatozoa (Castellani-Ceresa et al., 1992; Delgado-Buenrostro et al., 2005; Oikonomopoulou et al., 2009; Sanchez-Gutierrez et al., 2006). Meanwhile, inhibition of FAK prevented the actin polymerization and the co-localization of vinculin and α-actinin with F-actin (Figs 8 and 9), suggesting that FAK may drive the remodeling of the actin cytoskeleton. This remodeling involves the polymerization of actin in the acrosomal region and its association with the plasma membrane, most likely to avoid early loss of the acrosome (Spungin et al., 1995).

The increase in the co-localization of α-actinin and F-actin (Fig. 9) may suggest that α-actinin increases cross-linking of actin filaments. Nevertheless, we could not confirm an interaction between α-actinin and the focal adhesion proteins because α-actinin was co-immunoprecipitated in very small amounts with paxillin. This outcome may be explained by the strong association shown by α-actinin with actin filaments rather than with focal adhesion complexes (Kanchanawong et al., 2010). Likely the interaction of actin filaments with focal adhesion complexes is lost during co-immunoprecipitation processing, restricting the co-immunoprecipitation of α-actinin with paxillin.

Protein Tyr phosphorylation is a determinant event of capacitation (Aitken and Nixon, 2013). A recent study proposed that FAK is not involved in protein Tyr phosphorylation because when FAK is inhibited by PF537228, Tyr phosphorylation is not blocked (Alvau et al., 2016). Here, we obtained a similar result (Fig. 5A) but we also found that FAK inhibition has several other effects on the Tyr phosphorylation. Inhibition of FAK resulted in an early Tyr phosphorylation in the flagellum and the acrosome, as shown by the Tyr phosphorylation immunostaining (Fig. 6). This early increase in Tyr phosphorylation likely leads to an anticipated capacitation and therefore to an anticipated AR. We propose that the normal increase in Tyr phosphorylation during capacitation requires activation of FAK, where actin polymerization associated with capacitation is relevant. Three pieces of evidence support this suggestion: (1) FAK inhibition blocked actin polymerization (Fig. 7); (2) Lat-A, an important inhibitor of actin polymerization, caused an outcome similar to that observed by inhibition of FAK, Tyr phosphorylation was not blocked (Fig. 5A,B) and the AR increased (Fig. 5C); (3) previous work has also shown that the inhibition of actin polymerization during capacitation by cytochalasin D does not prevent capacitation or Tyr phosphorylation from proceeding normally (Bernabo et al., 2011). In conclusion, the adequate structuration of the actin cytoskeleton during capacitation leads to an ongoing and controlled Tyr phosphorylation, which might be part of the regulatory mechanism of PKA activity. Therefore, the blocking actin polymerization associated with capacitation, either by FAK inhibition or by drugs such as cytochalasin D and Lat-A, could lead to an increment in PKA activity and consequent early increase of Tyr phosphorylation. Interestingly, disruption of the actin cytoskeleton by cytochalasin D enhances cAMP formation and phosphorylation of PKA substrates in response to prostacyclin (Raslan and Naseem, 2015).

It is also important to consider that in contradistinction to humans and mice where Tyr phosphorylation is very low in non-capacitated spermatozoa (Battistone et al., 2014; Visconti et al., 1995), levels of Tyr phosphorylation are prominent in non-capacitated guinea pig spermatozoa, increase during capacitation, and are abolished by the PKA antagonist H89 (Fig. 5A). Perhaps PKA maintains a considerable amount of activity in non-capacitated guinea pig spermatozoa, such that tyrosine kinases remain active.

Our functional analysis of FAK inhibition shows two important effects on spermatozoa. First, those treated with a FAK inhibitor, such as PF573228, had an accelerated Ca^2+^-dependent AR. The second affect associates an early AR with the inhibition of actin polymerization, which in somatic cells is critical for controlling exocytosis processes; actin filaments create a barrier that prevents membrane fusion. In mammalian spermatozoa, this barrier is formed during capacitation (Breitbart et al., 2005; Cabello-Agueros et al., 2003; Spungin et al., 1995) keeping the acrosome integral to its encounter with the egg. We suggest that inhibiting FAK altered actin polymerization (Fig. 7), preventing the remodeling of the actin cytoskeleton in the apical acrosome region (Figs 8 and 9) to the point that the barrier of filamentous actin is not produced. As a consequence, the intracellular concentration of Ca^2+^ increases during capacitation allowing early sperm membrane fusion and therefore the AR. This hypothesis is supported by the inhibition of actin polymerization by Lat-A, which also increased the AR after 60 min of capacitation (Fig. 5C). Potentially, Lat-A takes longer to disrupt actin filaments than does inhibition of FAK. FAK is a kinase that is involved in actin polymerization, cytoskeleton remodeling and the formation and disassembly of cell adhesion structures. In addition, FAK regulates the activity of the Rho-family of GTPases, such as RhoA (Schaller, 2010; Tomar and Schlaepfer, 2009), a protein involved in the polymerization of actin (Ridley, 2006) that has been found in mammalian spermatozoa (Delgado-Buenrostro et al., 2005; Ducummon and Berger, 2006) and was recently related to actin polymerization in mouse spermatozoa (Romarowski et al., 2015). Recent results from our laboratory show that RhoA is activated during capacitation and that FAK inhibition by PF573228 prevented its activation (data not shown).

One question that emerges from these results is how FAK is activated during capacitation. FAK is a non-receptor tyrosine kinase that gets recruited to clustered integrin proteins. Canonical models postulate that integrins activate FAK (via the autophosphorylation of Tyr397) in response to integrins binding to ECM proteins, such as fibronectin, collagen, or laminin (Lawson and Schlaepfer, 2012). Because capacitation was performed *in vitro* and in a medium absent of ECM proteins, we propose that ECM proteins associated with the spermatozoa could be involved in the activation of FAK. Fibronectin, an important ECM protein, has been found in the heads of spermatozoa, particularly in the equatorial segment of human, porcine, bovine, and ram (Ekhlasi-Hundrieser et al., 2007; Leahy et al., 2011; Thys et al., 2009; Vuento et al., 1984). These observations, combined with the knowledge that spermatozoa interact through their head region during *in vitro* capacitation, cause us to suggest that these interactions facilitate the binding of fibronectin and integrins that ultimately activate FAK.

In summary, we have demonstrated that several proteins associate with the focal adhesion complex (FAK, paxillin, talin, and vinculin) in mammalian spermatozoa and that it is necessary to maintain acrosome integrity. Our results also show that these proteins form focal adhesion complexes in different regions of the spermatozoa that are important for capacitation and the AR. Interestingly, the complex formed in the acrosomal region matures during capacitation. The presence of FAK in mammalian spermatozoa is significant for sperm physiology because this kinase is critical for activating several signaling pathways that are potentially involved in capacitation and the regulation of cytoskeleton remodeling that occurs during capacitation. Further studies are necessary to determine how FAK and molecules associated with the focal adhesion complex participate in the cellular and molecular events that modulate capacitation and the AR. Finally, evidence of FAK in mammalian spermatozoa opens the potential for additional investigation into sperm physiology.

## MATERIALS AND METHODS

### Chemicals

Sodium pyruvate, lactic acid, DL-dithiothreitol, sucrose, Triton X-100, iodoacetamide, benzamidine, aprotinin, leupeptin, pepstatin, p-aminobenzamidine, phenylmethylsulfonyl fluoride (PMSF), Trizma base, FITC- or TRITC-phalloidin, sodium orthovanadate, and sodium fluoride were purchased from Sigma Chemical Co. (St. Louis, MO). Protein A/G-agarose and protease inhibitors (Complete™ cocktail tablets) were obtained from Roche Diagnostics and Molecular Biochemicals (Mannheim, Germany). Nitrocellulose membranes, acrylamide, N,N′-methylene-bis-acrylamide and sodium dodecyl sulfate (SDS) were obtaining from Bio-Rad Laboratories (Hercules, CA). Immobilon membranes were purchased from Millipore (Billerica, MA). Anti-FAK (sc-558, sc-1688), anti-paxillin (sc-5574), anti-vinculin (sc-7648 and sc-5573), anti-integrin-β1 (sc-6622 and sc-8978), anti-p-FAK (sc-11765-R), α-actinin (sc-15335), and anti-p-paxillin (sc-365020) were purchased from Santa Cruz Biotechnology Inc. (Santa Cruz, CA). Anti-Talin (T3287) and α-actinin (A-2543) were obtained from Sigma Chemical Co. (St. Louis, MO.) Secondary antibodies labeled with horseradish peroxidase (HRP), TRITC, FITC, or Cy5 were obtained from Jackson Immunoresearch Laboratories Inc. (West Grove, PA). Enhanced chemiluminescence (ECL) reagent was purchased from Amersham (Buckinghamshire, UK) or Millipore (Billerica, MA). The FAK inhibitor PF573228 (Cat. No. 3239) was obtained from Tocris Bioscience (Bristol, BS11, UK).

### Animals

All animal experiments and handling procedures were approved by the Internal Committee for Laboratory Animal Care and Use of the CINVESTAV-IPN (CICUAL 012211) following the American Veterinary Medical Association guidelines. Every effort was made to minimize the potential for animal pain, stress, or distress.

### Guinea pig spermatozoa preparation

Non-capacitated and capacitated spermatozoa were obtained as described previously (Cabello-Agueros et al., 2003; Pasten-Hidalgo et al., 2008). Briefly, guinea pig spermatozoa were obtained from the ductus deferens and washed in 154 mM NaCl solution. Sperm cells (3.5×10^7^ cell/ml) were capacitated by incubation at 37°C in minimal culture medium containing lactate and pyruvate (MCM-PL). Cells were incubated under capacitation conditions in the presence of PF57228 (0-10 µM) for 20 to 60 min in MCM-PL. Immediately after capacitation, samples were fixed in a 1.5% formaldehyde (final concentration) PBS solution. Depending on the experiment, control cells were incubated in a medium that did not support capacitation (MCM-PL without NaHCO_3_ and CaCl_2_, buffered with 25 mM HEPES, pH 7.4), MCM-PL, or MCM-PL plus a vehicle. In all cases, the control cells were incubated and fixed at the same time as the treated samples.

### Preparation of mouse spermatozoa

Non-capacitated and capacitated spermatozoa were obtained according to Hernández-González et al. (2006). In most experiments, caudal epididymal mouse sperm were collected from CD1 retired male breeders by placing minced caudal epididymis in a modified Krebs-Ringer medium [Whitten's HEPES-buffered (WH) medium]. This medium does not support capacitation and was first prepared in the absence of bovine serum albumin and NaHCO_3_. After 5 min, spermatozoa in the suspension were washed with 10 ml of the same medium by centrifugation at 800×***g*** for 10 min at room temperature. The sperm were then resuspended to a final concentration of 2×10^7^ cells/ml and diluted 10 times in the appropriate medium. In experiments where capacitation was investigated, 5 mg/ml of bovine serum albumin and 24 mM NaHCO3 were added. The pH was maintained at 7.6.

### Electrophoresis and immunoblotting

Cells (350×10^6^) were suspended in lysis buffer (50 mM Tris–HCl at pH 7.4, 1 mM EGTA, 1 mM PMSF, complete protease inhibitor cocktail, 1 mg/ml aprotinin, 10 mM sodium orthovanadate, 25 mM sodium fluoride, and 1% Triton X-100) as previously described (Pasten-Hidalgo, et al., 2008). The samples were centrifuged at 5000×***g*** for 5 min at 4°C, and the protein concentrations of the supernatant fractions were determined as described previously (Bradford, 1976). The samples were then boiled for 5 min in sample buffer (Laemmli, 1970), resolved on 7% or 10% SDS-PAGE gels and transferred onto PVF or nitrocellulose membranes (Towbin et al., 1979). The membranes were blocked using Tris-buffered saline (TBS) containing 0.1% Tween-20 and 5% fat-free dry milk. The membranes were incubated overnight at 4°C with the respective antibody (anti-FAK 1:100, anti-vinculin 1:1000, anti-paxillin 1:1000, anti-talin 1:1000, anti-integrin-β 1:1000, or anti-α-actinin 1:1000). The membranes were washed five times for 7 min each time and were then incubated with the appropriate HRP-labeled secondary antibody (1:10,000). Immunoreactive proteins were detected by chemiluminescence using an ECL western blot detection kit (Amersham or Millipore). Three types of controls were performed to verify specificity of the antibodies: (1) immunoblots with secondary antibody alone, (2) immunoblots with a non-specific primary antibody, and (3) primary antibodies pre-incubated with their respective blocking peptides prior to immunoblotting. Under these conditions, protein bands were either not observed or drastically reduced (Fig. S2B).

### Immunofluorescence procedures

Cells were fixed in 1.5% formaldehyde in PBS, permeabilized with acetone at −20°C for 7 min, and washed three times in PBS and once in distilled water. Water-resuspended cells were used to prepare smears, which were air-dried at room temperature and rinsed with PBS. The smears were then incubated with primary antibody (anti-FAK 1:100, anti-vinculin 1:100, anti-paxillin 1:100, anti-talin 1:100, anti-integrin-β 1:100, or anti-α-actinin 1:100) in blocking solution (1% bovine serum albumin in PBS) for 12 h at 4°C. After extensive washes with PBS, the cells were incubated with the appropriate TRITC- or Cy5-labeled secondary antibodies for 1 h at 37°C. In all cases, smears were extensively washed with PBS and mounted on cover glass slides using gelvatol (Sigma Chemical Co., St. Louis, MO) (Harlow and Lane, 2006). To confirm the specificity of the antibodies and the location of the focal adhesion proteins, three types of controls were assayed: (1) spermatozoa were only incubated with the secondary antibody, (2) spermatozoa were incubated with a non-specific primary antibody, and (3) the primary antibodies used were pre-incubated with their respective blocking peptides before the immunofluorescence assay. Spermatozoa stained under these conditions showed very faint patterns of fluorescence (Fig. S3).

### Co-immunoprecipitation

For the immunoprecipitation assays, the anti-paxillin antibody was bound to protein A/G-agarose according to the manufacturer's instructions (Pierce^®^ Crosslink Immunoprecipitation Kit). Briefly, 20 µg of each antibody was coupled to 20 µl of the resin, incubated for 1 h at room temperature and then washed to eliminate excess antibodies. Crosslinking was achieved by incubating the bound antibody in the presence of disuccinimidyl suberate at room temperature for 60 min and washing to eliminate excess non-crosslinked antibody. The sperm protein extracts from non-capacitated and capacitated spermatozoa were then incubated with constant agitation for 12 h at 4°C. The proteins bound to the A/G-agarose antibody were recovered by centrifugation at 4000×***g*** after three washes. The associated proteins were eluted and recovered by centrifugation at 4000×***g*** and boiled in Laemmli sample buffer (Bio-Rad Laboratories, Hercules, CA) for 5 min. Sperm proteins were separated by SDS-PAGE, transferred to nitrocellulose membranes, and subjected to immunoblot analysis.

### Protein tyrosine phosphorylation

Spermatozoa (350×10^6^) were resuspended in lysis buffer (50 mM Tris–HCl at pH 7.4, 1 mM EGTA, 1 mM PMSF, complete protease inhibitor cocktail, 1 mg/ml aprotinin, 10 mM sodium orthovanadate, 25 mM sodium fluoride, and 1% Triton X-100) as previously described (Pasten-Hidalgo, et al., 2008). The samples were centrifuged at 5000×***g*** for 5 min at 4°C, and the protein concentrations of the supernatant fractions were determined as described previously (Bradford, 1976). 100 µg of each sample was boiled for 5 min in sample buffer (Laemmli, 1970), resolved on 10% SDS-PAGE gels, and transferred onto nitrocellulose or PVF membranes (Towbin et al., 1979). The membranes were blocked using TBS containing 0.1% Tween-20 and 5% bovine serum albumin. The antibodies against phosphor-tyrosine (anti-p-Tyr) were diluted (1:1000) in the blocking solution and added to the membranes and incubated overnight at 4°C. The membranes were washed five times for 7 min each time and then incubated with the appropriate HRP-labeled secondary antibody (1:10,000). Immunoreactive proteins were detected by chemiluminescence using an ECL western blot detection kit (Amersham or Millipore).

### Evaluation of capacitation status by staining with chlortetracycline (CTC)

We followed the method described by Shi and Roldan (1995) to prepare spermatozoa for the CTC assessment. In short, the stain solution was prepared by dissolving 250-µM CTC-HCl in TN buffer (20 mM Tris, 130 mM NaCl and 5 mM cysteine at pH 7.8); fresh CTC stock was prepared daily. Sperm suspensions (20 µl) were added to the same volume of pre-warmed (37°C) CTC stock solution and incubated for 10 s and 3.5 µl of 12.5% glutaraldehyde in 1.25 M Tris buffer (pH 7.5) was added, immediately followed by gentle mixing. Fixed samples were kept in a dark box. Slides were prepared 1-4 h after fixation and examined under a fluorescence microscope (excitation at 330-380 nm, emission at 420 nm). A total of 100 spermatozoa were counted to assess the previously recognized different CTC staining patterns (Ward and Storey, 1984): F=non-capacitated, B=capacitated with an intact acrosome, and AR=undergone acrosomal exocytosis.

### Assessment of F-actin

Spermatozoa were fixed in 1.5% formaldehyde in PBS for 1 h at room temperature and collected by centrifugation at 600×***g*** for 4 min. The sperm pellet was immediately resuspended and incubated in 50 mM NH_4_Cl in PBS for 10 min and washed twice by resuspension/centrifugation in PBS. Sperm smears were prepared and used for staining. Spermatozoa on smears were permeabilized using acetone at 20°C for 7 min and washed in PBS. F-actin was revealed using TRITC-phalloidin, and spermatozoa were incubated with 30-µM TRITC-phalloidin at room temperature for 60 min. The smears were exhaustively washed with PBS and mounted on glass covers for observation using gelvatol. Images were acquired using an Olympus BX50 photomicroscope equipped with phase contrast and epifluorescence or using a Leica confocal microscope. Fluorescence was analyzed using Nikon Nis 2.0 software.

### Determination of the optimal concentration of PF573228

To investigate the possible participation of FAK in capacitation or the AR, in this work we used the FAK antagonist PF573228, a specific FAK inhibitor (Cabrita et al., 2011; Jones et al., 2009; Slack-Davis et al., 2007; Tse et al., 2012). Spermatozoa were capacitated in the presence of different concentrations of PF5732228 (0.5-10 µM) and after 20 min the AR was quantified. The maximum effect was observed between 2.5-5.0 µM PF5732228 (Fig. S4B). Note that although spermatozoa lost their acrosome, they maintained their motility (data not shown).

### Sperm viability assay

Since the effects of PF573228 on mammalian spermatozoa were unknown and because of the acrosome loss it induced, we were motivated to evaluate the viability of these spermatozoa. Sperm viability was estimated by assessing the membrane integrity of the cell using propidium iodide (PI) dye exclusion, according to Brito et al. (2003). We added a PI solution (1 µg/ml) to a sample of spermatozoa to a 1:1 ratio and mixed and incubated the mixture at room temperature for 30 min. The spermatozoa were washed and the number of stained and unstained spermatozoa was counted (500 cell×sample, *n*=3) under an epifluorescence microscope. Spermatozoa capacitated in the absence of PF573228 after 60 min of capacitation showed a 96.33% ±0.84 (means±s.e.m., *n*=3) of viability, while spermatozoa capacitated in presence of 10 µM PF573228, the highest concentration tested, showed no significant difference in viability, 95.67%±1.46 (means± s.e.m., *n*=3) (Fig. S4A,B).

### Estimating the AR

We estimated the AR for guinea pig sperm by examining small drop samples under light microscopy (Yanagimachi and Bhattacharyya, 1988). After a predetermined time of capacitation (20, 60, or 90 min) spermatozoa were fixed (formaldehyde 1.5%, final concentration). The AR was quantified based on the percentage of spermatozoa without an acrosome before and after capacitation. Four aliquots were separately counted (300 cells each) in a Neubauer chamber. In capacitated spermatozoa, the AR was normalized with respect to the acrosome loss of non-capacitated sperm; the number of spermatozoa without acrosome, before capacitation, was subtracted from the number of spermatozoa that experienced RA during capacitation.

### Cell culture and lysis

The human MDA-MB-231 breast cancer cell line was cultured using Dulbecco's modified Eagle's medium supplemented with 3.7 g/l of sodium bicarbonate and 5% fetal bovine serum. They were kept at 37°C under a humidified atmosphere containing 5% CO_2_ and 95% air. When cells were confluent, the medium was eliminated by aspiration and cells were solubilized in RIPA buffer (50 mM HEPES at pH 7.4, 150 mM NaF, 10 mM sodium pyrophosphate, 10% glycerol, 1% Triton X-100, 1% sodium deoxycholate, 1.5 mM MgCl_2_, 0.1% SDS, and protease inhibitors). Cell lysates were clarified by centrifugation at 22,500×***g*** for 10 min at 4°C. Supernatants were transferred to fresh tubes and protein levels were determined by Bradford protein assay.

### Statistical and data analyses and densitometric analysis

Data are expressed as means±s.e.m. Means were compared using an unpaired Student's *t*-test or Student's *t*-test as appropriate. The statistical significance between the different samples was considering with a significance of *P*<0.05. Fluorescence data were normalized with respect to the results of non-capacitated spermatozoa and set at 1, and to obtain the fluorescence increases, the ratios of capacitated:non-capacitated were obtained and expressed as an increase in fluorescence. Densitometric analyzes were performed using ImageJ1 software (Research Services Branch of the NIH). For focal adhesion complex formation (Fig. 3A,B) and the results are expressed as the ratio *N*:No, where *N* is the total amount of each co-immunoprecipitated protein and No is the total amount of paxillin immunoprecipitated. For FAK and paxillin phosphorylation (Fig. 4), as well as Tyr phosphorylation (Fig. 6B), protein phosphorylation was expressed as the ratio *N*:No, where *N* is the total amount protein phosphorylated for each time of capacitation and treatment and No is the total amount of phosphorylated proteins for non-capacitated spermatozoa.
